# Solar Water Disinfection in Household Settings: Hype or Hope?

**DOI:** 10.1371/journal.pmed.1000127

**Published:** 2009-08-18

**Authors:** Zulfiqar A. Bhutta

**Affiliations:** Division of Women & Child Health, The Aga Khan University, Karachi, Pakistan

## Abstract

Zulfiqar Bhutta discusses a new trial of solar water disinfection in rural Bolivia, and questions whether such technologies offer just hype or new hope for communities struggling with unclean water and poor sanitation.

**Linked Research Article**

This Perspective discusses the following new study published in *PLoS Medicine*:

Mäusezahl D, Christen A, Duran Pacheco G, Alvarez Tellez F, Iriarte M, et al. (2009) Solar Drinking Water Disinfection (SODIS) to Reduce Childhood Diarrhoea in Rural Bolivia: A Cluster-Randomized, Controlled Trial. PLoS Med 6: e1000125. doi:10.1371/journal.pmed.1000125

Daniel Mäusezahl and colleagues conducted a cluster-randomized controlled trial in rural Bolivia of solar drinking water disinfection, and find only moderate compliance with the intervention and no evidence of reduction in diarrhea among children.

Millennium Development Goal (MDG) 7, and its target 3, aims to halve the proportions of people globally without sustainable access to safe drinking water and basic sanitation by 2015 [1]. Others have argued that access to safe drinking water is a fundamental human right [2]. Several interventions to improve access to clean water and sanitation, including one reported in this week's *PLoS Medicine* [3], have been evaluated and found to have varying degrees of success. As such, the key question remains unanswered: do water and sanitation interventions offer hype or hope?

## Water, Sanitation, and Hygiene (WASH) Interventions

In assessing the potential impact of various interventions on reducing child mortality in 2003, we had previously estimated that provision of improved water, sanitation, and hygiene (WASH) interventions at universal coverage could avert 3% of the burden of child mortality [4]. Other estimates by the World Health Organization suggest that up to 28% of under-five deaths could be attributable to poor sanitation and unsafe water, and that WASH interventions have the potential to prevent 25% of the overall under-five disease burden (morbidity and mortality) [5]. In a recent review of interventions to reduce child undernutrition, we estimated that WASH interventions could reduce the incidence of diarrhea by 30% and related stunting (an estimated 4% increase in the odds of stunting with each episode of diarrhea) [6].

These burden and intervention effect estimates are not mirrored by a consensus on what needs to be done to address these issues at scale and whether the respective WASH interventions should be administered singly or as a package. In an earlier analysis, Esrey et al. [7] had suggested that the impact of multiple WASH interventions on reducing childhood diarrhea was no greater than single interventions implemented alone. In a more recent meta-analysis [8], Fewtrell et al. evaluated 46 studies with WASH interventions to assess their impact on childhood diarrhea. Their pooled analysis also suggested that water quality interventions (such as point-of-use water treatment) were effective in reducing diarrhea by 31% (95% confidence intervals [CI] 0.53–0.89), but multiple interventions (consisting of combined water, sanitation, and hygiene measures) were of comparable benefit to single interventions. These findings may be clearly influenced by variations in context and trial design. More recent data from a study following the introduction of a city-wide urban sanitation program in Salvador, Brazil [9], indicated that improvement in sanitation coverage alone in the wake of almost universal safe water availability was associated with a reduction in diarrhea prevalence by 21% (95% CI 18%–25%). These data suggest that the effect of various WASH interventions may be potentially additive.

The potential costs of various component WASH interventions are clearly not comparable. The estimated costs of improved water supply and excreta disposal for at least five years in settings where established infrastructure currently exists are over US$4,185 per disability-adjusted life year averted [10]. Other behavioral change hygiene interventions, though less expensive, may have limited long-term effectiveness. To illustrate, while promotion of hand washing has been shown to significantly reduce the burden of diarrhea in community settings [11], the effect is difficult to sustain over time [12]. There is thus considerable interest in interventions to improve water quality, and Clasen et al. [13] recently reviewed 33 studies of interventions to improve water quality and their impact on childhood diarrhea. Their review shows significant heterogeneity among available studies and considerable contextual differences. The two studies of solar water disinfection (SODIS) from Kenya [14] and India [15] were associated with significantly reduced odds of diarrhea among children under five (odds ratio 0.69, 95% CI 0.63–0.74).

## New Evidence on Solar Disinfection (SODIS)

The study by Mäusezahl and colleagues in rural Bolivia [3] is the largest cluster-randomized controlled trial (RCT) to date of SODIS promotion at a population level and demonstrates the difficulty in scaling up such interventions in health systems. Despite relatively intense promotion of the intervention through community workers from a local nongovernmental organization, the effect on diarrhea incidence in the intervention clusters was insignificant (RR 0.81, 95% CI 0.59–1.12). It is uncertain if the trial reflects a failure of the intervention as opposed to the delivery strategy itself. The compliance achieved with the intervention itself was a mere 32.1%, and hence the observed effect size probably reflects the overall poor coverage of the intervention. It should also be underscored that the sample size was based on the effect size estimated from much smaller efficacy studies, and that the trial was significantly underpowered to observe lower but still highly relevant impacts.

It is important to emphasize the inherent complexity of behavioral changes and household practices research in effectiveness settings. The failure of some plausible interventions when implemented at scale may also reflect a failure of delivery strategies rather than an ineffective intervention. It is understandable as to why the investigators chose a cluster RCT design, rather than alternative strategies such as a stepped wedge design [16], although the latter might have arguably been more suitable for the phased implementation and evaluation of such interventions. It should also be pointed out that despite the dispersed population clusters, the nature of the study design would have precluded the use of mass media and other concerted promotional strategies in this setting. The intervention was implemented through a combination of bi-weekly domiciliary and monthly community meetings, although it is unclear what levels of community participation were achieved in the latter. It is possible that alternative methods of community engagement, such as use of community support groups, might have been more effective. These approaches have been successfully employed for maternal and newborn care interventions in a variety of community settings [17],[18] and are especially relevant for behavioral change at a household level.

Notwithstanding the inherent difficulties in translating efficacy data from biologically plausible interventions into effectiveness settings, we applaud such research, as it is precisely the type of information needed to facilitate delivery of key interventions to address the MDGs. Mäusezahl et al. are right in calling for further research on the effectiveness of SODIS in population settings, a point also underscored by findings from the follow-up of a household water treatment and hand washing promotion intervention in rural Guatemala [19]. It is also important, however, to highlight the need to complement such research with appropriate and robust evaluation of delivery strategies and approaches to promote community buy-in. The observed direction of effect on childhood diarrhea in the Bolivia study is encouraging and supports further evaluation of low-cost and sustainable interventions to promote point-of-use water purification techniques in rural communities.
